# Mechanisms of Siglec-F-Induced Eosinophil Apoptosis: A Role for Caspases but Not for SHP-1, Src Kinases, NADPH Oxidase or Reactive Oxygen

**DOI:** 10.1371/journal.pone.0068143

**Published:** 2013-06-28

**Authors:** Hui Mao, Gen Kano, Sherry A. Hudson, Mary Brummet, Nives Zimmermann, Zhou Zhu, Bruce S. Bochner

**Affiliations:** 1 Division of Allergy and Clinical Immunology, Department of Medicine, The Johns Hopkins University School of Medicine, Baltimore, Maryland, United States of America; 2 Division of Allergy and Immunology, Cincinnati Children’s Hospital Medical Center and University of Cincinnati College of Medicine, Cincinnati, Ohio, United States of America; Fundação Oswaldo Cruz, Brazil

## Abstract

**Background:**

Siglec-F and Siglec-8 are functional paralog proapoptotic cell surface receptors expressed on mouse and human eosinophils, respectively. Whereas Siglec-8 mediated death involves caspases and/or reactive oxygen species (ROS) generation and mitochondrial injury, very little is known about Siglec-F-mediated signaling and apoptosis. Therefore the objective of the current experiments was to better define apoptosis pathways mediated by Siglec-F and Siglec-8. Given that Siglec-F-induced apoptosis is much less robust than Siglec-8-induced apoptosis, we hypothesized that mechanisms involved in cell death via these receptors would differ.

**Methods:**

Consequences of engagement of Siglec-F on mouse eosinophils were studied by measuring ROS production, and by performing apoptosis assays using eosinophils from normal, hypereosinophilic, NADPH oxidase-deficient, src homology domain-containing protein tyrosine phosphatase (SHP)-1-deficient, and Lyn kinase-deficient mice. Inhibitors of caspase and Src family kinase activity were also used.

**Results:**

Engagement of Siglec-F induced mouse eosinophil apoptosis that was modest in magnitude and dependent on caspase activity. There was no detectable ROS generation, or any role for ROS, NADPH oxidase, SHP-1, or Src family kinases in this apoptotic process.

**Conclusions:**

These data suggest that Siglec-F-mediated apoptosis is different in both magnitude and mechanisms when compared to published data on Siglec-8-mediated human eosinophil apoptosis. One likely implication of this work is that models targeting Siglec-F *in vivo* in mice may not provide identical mechanistic predictions for consequences of Siglec-8 targeting *in vivo* in humans.

## Introduction

Eosinophils have been implicated in a variety of disorders ranging from asthma and allergic diseases to helminth parasite immunity to hematologic disorders [1], [2]. Biologics that target eosinophils by neutralizing the major eosinophil hematopoietic cytokine IL-5, or target the IL-5 receptor to facilitate eosinophil depletion, are advancing in clinical trials [3]–[7].

Siglecs (sialic acid-binding, immunoglobulin-like lectins) are a family of single-pass transmembrane cell surface proteins found predominantly on leukocytes [8], [9]. Siglec-8 was discovered as part of efforts initiated about a decade ago to identify novel human eosinophil proteins; subsequent studies also detected its expression on mast cells [10]–[12]. The closest functional paralog in the mouse is Siglec-F, which is also selectively expressed by eosinophils but not on mast cells [12]–[15]. Both Siglec-8 and Siglec-F preferentially recognize the glycan 6′-sulfo-sialyl Lewis X (6′-sulfo-sLe^X^) [16]–[18], and studies in mice implicate a sialylated glycoprotein made by airways epithelium as an endogenous ligand [19]–[21]. Engagement of Siglec-8 or Siglec-F with Abs and/or artificial ligands in vitro causes eosinophil death [18], [19], [22]–[24].

Mechanistic studies with Siglec-8 implicated both caspases and reactive oxygen species (ROS) generation resulting in mitochondrial injury in eosinophil death [25]. Interestingly, Siglec-8-induced cell death could not be overridden by survival-inducing cytokines such as IL-5, GM-CSF and IL-33. In fact, Siglec-8-induced eosinophil apoptosis was enhanced by these cytokines [22], [23], [26], [27]. Similar results were obtained using human eosinophils primed in vivo following allergen bronchoprovocation, and primed cells no longer used caspases in the apoptosis process, instead relying exclusively on ROS generation and mitochondrial injury [26].

Most siglecs, including Siglec-8 and Siglec-F, have immuno-receptor tyrosine-based inhibitory motifs (ITIMs) in their intracellular domain, suggesting an inhibitory role for signaling [8], [9], [28], [29]. The membrane proximal motif contains a well-recognized 6-amino acid sequence described as (I/L/V)xYxx(L/V) [30] that would putatively act with src homology domain-containing protein tyrosine phosphatases (SHP) such as SHP-1 following phosphorylation by Src kinases, such as Lyn [31], [32]. Indeed, ITIMs in various siglecs can recruit SHP-1 when phosphorylated [33], and modulate cellular activity in an inhibitory manner upon cross-linking with antibodies [34]. The function of the membrane distal CD150-like tyrosine motifs found in both Siglec-8 and Siglec-F are less well explored, but it has been suggested that such motifs could represent immuno-receptor tyrosine-based switch motifs (ITSMs) that could mediate either inhibitory or activating signals [29], [35].

Conclusions from studies of Siglec-F have generally paralleled those of Siglec-8. For example, antibody cross-linking of Siglec-F on mouse eosinophils causes apoptosis [24], [36], [37], albeit not as robust as has been seen with human eosinophils via Siglec-8 [22], [25]. Administration of Siglec-F antibodies in mouse models of chronic allergic asthma and blood and gastrointestinal eosinophilia normalizes inflammatory responses and abrogates many aspects of tissue remodeling [24], [36]–[38]. Separate from effects on cell survival, many siglec proteins undergo endocytosis following engagement in an ITIM-dependent manner [39]. For example, Siglec-F becomes internalized when engaged with ligands, and internalization was dependent on its cytoplasmic ITIM motif [40]. However, the exact pathways of signaling for Siglec-8 and Siglec-F are still incompletely understood.

Given the potential similarities and differences involved in Siglec-8 and Siglec-F mediated apoptosis, the goal of this work was to define apoptosis pathways mediated by Siglec-F and explore the role of ROS, NADPH oxidase activity, SHP-1 and caspases involved in Siglec-F-induced cell death. Our data suggest that at least for studies of eosinophil apoptosis, examination of Siglec-F does not always provide the same mechanistic conclusions as for Siglec-8.

## Materials and Methods

### Mice

All experiments were performed in accordance with the National Institutes of Health guidelines for the humane treatment of animals, and were approved by the Johns Hopkins Institutional Animal Care and Use Committee under protocol #MO10M417 and the Cincinnati Children’s Hospital Medical Center Institutional Animal Care and Use Committee under protocol #OD11085. All mice were of the C57BL/6 background strain. (CD3d-IL5)NJ.1638Nal transgenic mice (IL-5^+/+^ mice) displaying splenomegaly and marked blood eosinophilia [41] were kindly provided by Drs. James and Nancy Lee (Mayo Clinic, Scottsdale, AZ), while B6(Cg)-*Ncf1^m1J^*/J mice with defective NCF1/p47^phox^ protein, resulting in reduced NADPH oxidase activity and ROS production (Ncf^−/−^ mice), viable motheaten mice deficient in Src-homology 2-domain phosphatase (SHP)-1 (me^v^ (Ptpn6^me-v^) mice) and wild-type mice were obtained from The Jackson Laboratory (Bar Harbor, ME). For some experiments, (CD3d-IL5)NJ.1638Nal transgenic mice were backcrossed with B6(Cg)-*Ncf1^m1J^*/J mice and then interbred to create IL-5 transgenic mice with defective NCF1/p47^phox^ protein (Ncf/IL-5^0/+^ mice). Lyn-deficient mice were obtained from Dr. Juan Rivera (National Institutes of Health, Bethesda, MD) and wild type littermates were used as control. Depending on the purpose of the experiment, cells from 8–22 week old mice were used.

### Antibodies

Rat mAbs directed against murine FAS (CD95, clone RMF2, IgG1, Beckman Coulter, Brea, CA), Siglec-F (Clone E50–2440, rat IgG2a, BD Biosciences), CCR3 (Rat IgG2a, R&D Systems, Minneapolis, MN), and irrelevant isotype-matched rat mAb (BD Biosciences) were purchased from the sources indicated.

### Isolation of Mouse Eosinophils from Blood, Spleen and Peritoneal Lavage

Blood was obtained via cardiac puncture from IL-5^+^ and Ncf/IL-5^0/+^mice. Spleens were also simultaneously obtained and single cell suspensions were generated. Erythrocytes were then lysed hypotonically. This yielded eosinophils of purity ranging from 28% to 55%, with all contaminating cells being mononuclear cells. In some experiments, immunomagnetic negative selection of EDTA-anticoagulated whole blood buffy coats was performed to generate blood eosinophils of >96% purity by depleting mononuclear cells with a mixture of CD90.2 and CD45R coated beads (Miltenyi Biotec, Auburn, CA) as previously described [42]. In other experiments, eosinophils were obtained from the peritoneal cavity 48–72 hours after 4% thioglycollate injection, as described, with purity ranging from 25% to 62% (average 36.6%, SD 11.6%), the contaminating cells being neutrophils and monocytes [43].

### Culture-derived Generation of Eosinophils from Bone Marrow Precursors

Ex vivo generation by culture of mouse bone marrow-derived eosinophils was performed essentially as previously described by Dyer et al [44]. Briefly, bone marrow cells were collected from the femurs and tibias of wild-type, Ncf^−/−^ and me^v^ mice by flushing the bone marrow cavity with RPM 1640 medium (Gibco BRL, Grand Island, NY). After red cell lysis, the bone marrow cells were cultured at 10^6^/ml in medium containing RPMI 1640 with 20% fetal bovine serum (FBS, Cambrex, East Rutherford, NJ), 100 IU/ml penicillin, 10 µg/ml streptomycin, nonessential amino acids, 1 mM sodium pyruvate (all from Invitrogen-Life Technologies, Grand Island, NY), and 50 µM 2-ME (Sigma-Aldrich, St. Louis, MO) supplemented with 100 ng/ml stem cell factor (SCF) and 100 ng/ml FLT3 ligand (FLT3-L) (PeproTech, Rocky Hill, NJ) from days 0 to 4. On day 4, the cells were moved to new flasks and the medium containing SCF and FLT3-L was replaced with medium containing 10 ng/ml recombinant mouse IL-5 (R&D Systems, Minneapolis, MN). Every other day, from this point forward until cells were used, one-half of the media was replaced with fresh media containing IL-5, and the concentration of the cells was adjusted each time to 10^6^/ml. Cells were enumerated in a hemocytometer and viability (consistently >90%) and purity were determined as mentioned above for human eosinophils. These methods yield eosinophils of normal morphology expressing proteins seen in mature eosinophils, including normal cell surface levels of Siglec-F [44].

### Evaluation of Mouse Eosinophil Phenotype and Apoptosis

Binding of antibodies and appropriate isotype controls (all used at saturating or equivalent concentrations) was determined on eosinophils by use of single or dual color immunofluorescence and flow cytometry with appropriate gating (e.g., CCR3^+^ granulocytes) employing a FASCalibur flow cytometer (BD Biosciences) as described previously [24], [44].

Eosinophil apoptosis was assessed for freshly isolated cells or mature cells grown from bone marrow-derived cells using flow cytometry following labeling with Annexin-V as described [24]. Briefly, apoptosis-inducing antibodies (Siglec-F, FAS and other controls) were added and cells were incubated for 18–24 hr in 10 ng/ml mouse IL-5 (R&D Systems). In some experiments, cells were preincubated for 5–30 minutes with the pan-caspase inhibitor Z-VAD-FMK (carbobenzoxy-valyl-alanyl-aspartyl-[O-methyl]- fluoromethylketone, 10 µM), Src family kinase inhibitor PP2, its inactive control PP3, Src family/ABL tyrosine kinase inhibitor dasatinib or the ROS production inhibitor diphenyleneiodonium (DPI, 20 µM) (each obtained from EMD Chemicals, San Diego, CA except for dasatinib which was from Bristol-Myers Squibb, Princeton, NJ) before adding the proapoptotic antibodies as previously described [22], [25].

### Measurement of Eosinophil ROS Production

The release of ROS was quantified using a lucigenin-dependent, 96-well based chemiluminescence assay exactly as described [45]. Briefly, eosinophils (100,000/well) suspended in Hanks’ balanced salt solution (pH 7.4) containing 10 mM HEPES and 1 mg/ml bovine serum albumin were added to 96 well tissue culture plates. For some wells, 1 ng/ml phorbol myristate acetate (PMA, Sigma-Aldrich) was used as a positive control, while others contained 10 µg/ml anti-Siglec-F or control IgG2a rat antibody. Chemiluminescence readings were repeatedly determined in triplicate using a Veritas Microplate Luminometer (Turner Biosystems, Inc., Sunnyvale, CA) for up to 150 min at 37°C. Data are reported as chemiluminescence intensity (arbitrary units) per 10^5^ eosinophils.

### Statistical Analyses

All data are presented as means ± SD. Statistical analysis was conducted using ANOVA, followed by post-hoc multiple comparison tests. Values were considered significant at P<0.05.

## Results

**Figure 1:**
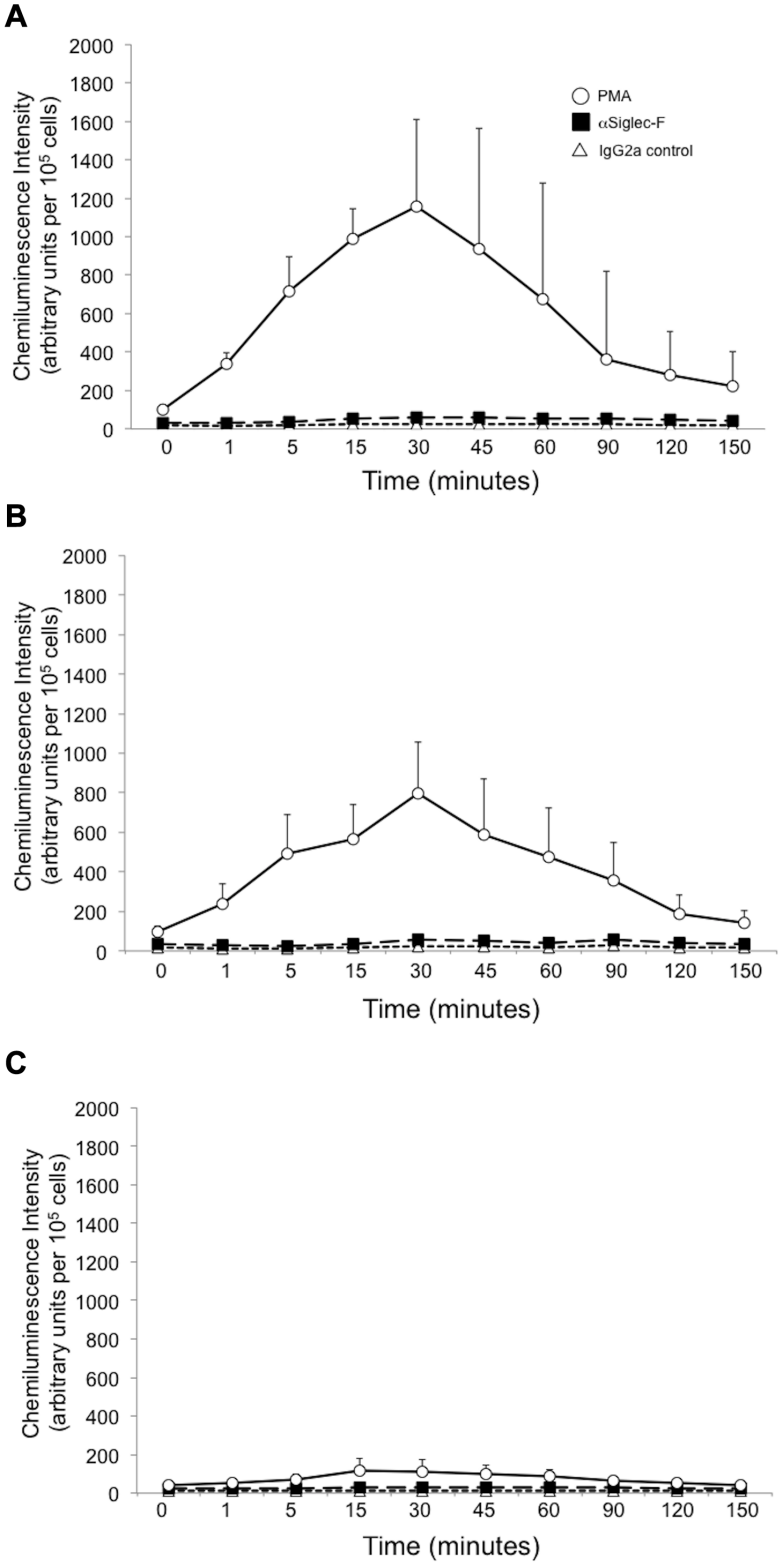
Exposure of mouse eosinophils to Siglec-F mAb does not result in detectable ROS production. Eosinophils were isolated from IL-5^+^ mice (panels A and B) or from Ncf/IL-5^0/+^ mice (Panel C) and exposed as indicated in the legend to Siglec-F mAb or an irrelevant isotype-matched rat IgG2a mAb. PMA (1 ng/ml) was used as the positive control. Panel A represents data using spleen cells (44±8% pure eosinophils, n = 4). Panel B represents data from peripheral blood (30±3% purity, n = 4). Panel C represents pooled data from two mice in which blood eosinophils and spleen eosinophils were tested separately with similar results, yielding a total of 4 separate experiments (34±12% purity).

Previous studies of Siglec-8-induced apoptosis using inhibitors of ROS generation in human eosinophils suggested that this pathway is particularly important in IL-5-activated human eosinophils [23], [26]. Therefore, the contribution of ROS to Siglec-F-induced murine eosinophil activation was initially explored. As shown in Figure 1, murine eosinophils isolated from the spleen or blood of IL-5^+^ mice produced ROS in response to PMA but not in response to Siglec-F mAb exposure (panels A and B). Furthermore, eosinophils isolated from the blood or the spleen of IL-5^+^ mice crossbred with the Ncf^−/−^ mice (so-called Ncf/IL-5^0/+^ mice) produced very little ROS, even in response to PMA treatment (panel C). This was not due to differences in Siglec-F surface expression among cell populations, because eosinophils from all sources used expressed similar surface levels of Siglec-F, and use of eosinophils cultured for up to 24 hours in the absence of IL-5 yielded similar results (data not shown). Furthermore, this was not due to an effect of contaminating cells, because other preparations containing blood eosinophils from IL-5 transgenic mice enriched to >96% purity also failed to produce any detectable ROS in response to anti-Siglec-F antibody, even though similar levels of ROS to those made by impure cells were detected in response to stimulation with PMA (data not shown).

**Figure 2:**
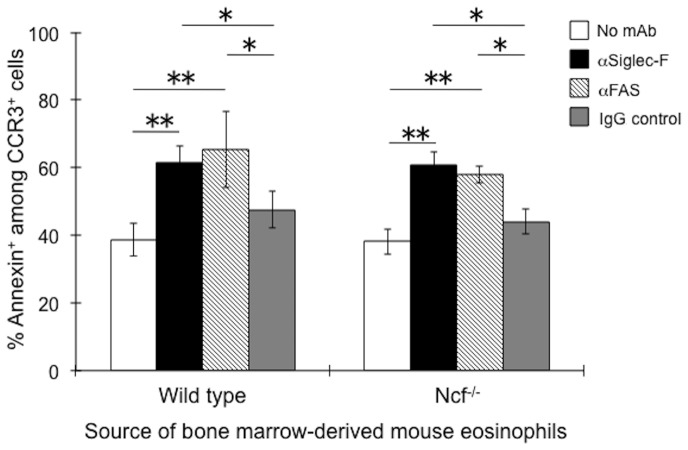
Eosinophils from Ncf^−/−^ mice undergo Siglec-F-induced apoptosis to the same degree as those of wild type mice. Eosinophils were grown from bone marrow precursors of wild type and Ncf^−/−^ mice. Purity of eosinophils at day 10 of culture was 52±23% and 50±22% for Ncf^−/−^ mice and wild type mice respectively (n = 4). Antibody exposure conditions are provided in the legend. Open bars: no mAb; black bars: Siglec-F mAb; striped bars: FAS mAb; gray bars: irrelevant rat IgG1 mAb (n = 2, to control for the FAS mAb) and irrelevant rat IgG2a mAb (n = 2, to control for the Siglec-F mAb). As no differences in effects were see with the two isotype-match control mAbs, data were pooled and shown as “IgG control”; n = 4. *p<0.05; **p<0.01.

Despite the lack of detectable ROS production following Siglec-F engagement, eosinophils grown from bone marrow-derived precursors (Figure 2), underwent apoptosis when exposed to either Siglec-F mAb or anti-FAS mAb as a control, and virtually identical levels of apoptosis were seen under these conditions, whether or not the eosinophils were derived from wild type, Ncf^−/−^ mice. Note that the magnitude of this modest apoptotic response was remarkably similar to that seen with identical methods employed with higher purity eosinophils derived from IL-5 transgenic mice as we and others have previously published, even when a secondary polyclonal anti-rat secondary antibody was used [19], [24]. These data suggest that the use of culture-derived eosinophils provides a suitable model for studying mechanisms of Siglec-F-mediated eosinophil apoptosis.

**Figure 3:**
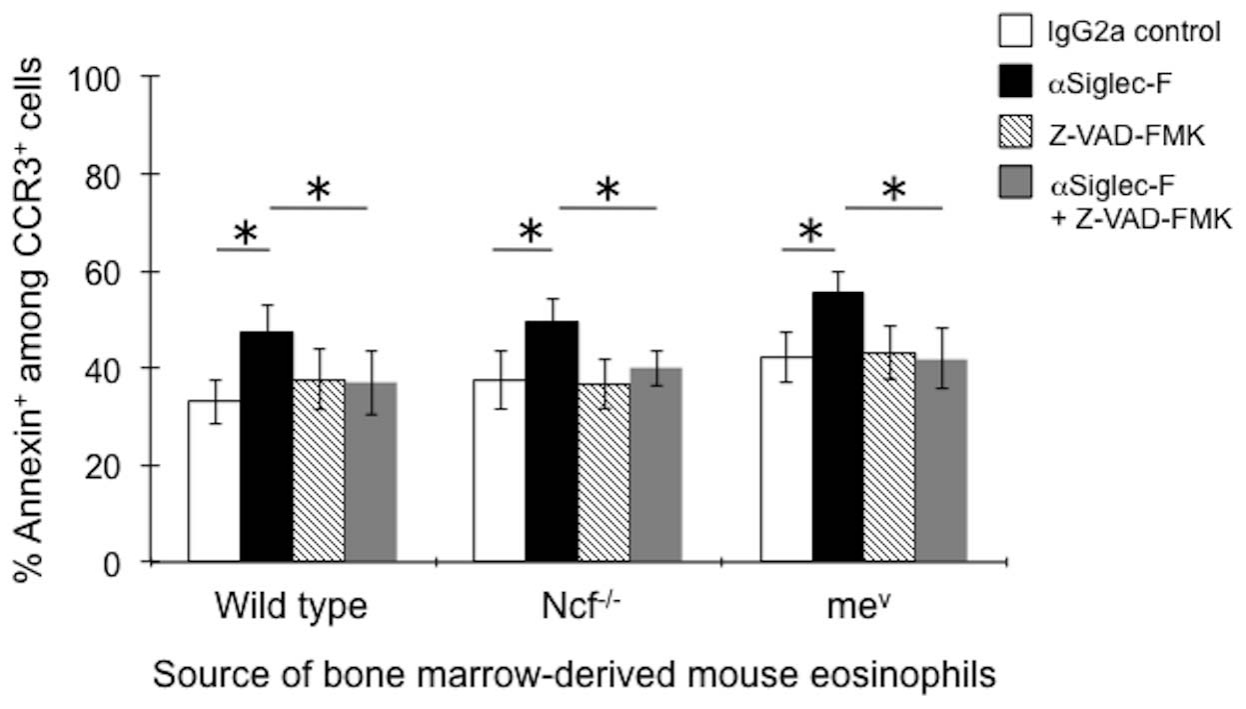
Effects of the pan-caspase inhibitor Z-VAD-FMK (10 µM) on Siglec-F-induced eosinophil apoptosis. Eosinophils were generated from bone marrow of wild type, Ncf^−/−^ and me^v^ mice and tested at day 10 of culture (purity was 46±20%, 52±18% and 41±23%, respectively, n = 3–6). Cells were preincubated for 30 minutes with or without Z-VAD-FMK followed by co-culture with Siglec-F or control irrelevant rat IgG2a mAb for an additional 18 hr before determining Annexin positivity. Various treatment conditions are provided by the legend. * p<0.05.

The next set of experiments was designed to directly explore the role of NADPH oxidase, SHP-1, Src family kinases and caspases in Siglec-F-induced eosinophil apoptosis. For these experiments, eosinophils were derived from the bone marrow of wild type, Ncf^−/−^ and me^v^ mice, the latter used to explore the role of SHP-1 in Siglec-F-mediated apoptosis. Apoptosis was determined in the presence or absence of Siglec-F mAb and the pan-caspase inhibitor Z-VAD-FMK. As shown in Figure 3, Siglec-F mAb was equally effective in inducing apoptosis among eosinophils derived from wild type, Ncf^−/−^ and me^v^ mice, and significant inhibition by Z-VAD-FMK of Siglec-F-induced apoptosis in all three types of mouse eosinophils was also seen, demonstrating that Siglec-F-induced apoptosis does not require NADPH oxidase activity or SHP-1 but is dependent on caspase activation. These results are in contrast to published work showing that human eosinophil apoptosis induced by Siglec-8 is only partially caspase-dependent, and after exposure to IL-5 or IL-33 primarily involves mitochondrial pathways and the generation of ROS [22], [25]–[27].

**Figure 4:**
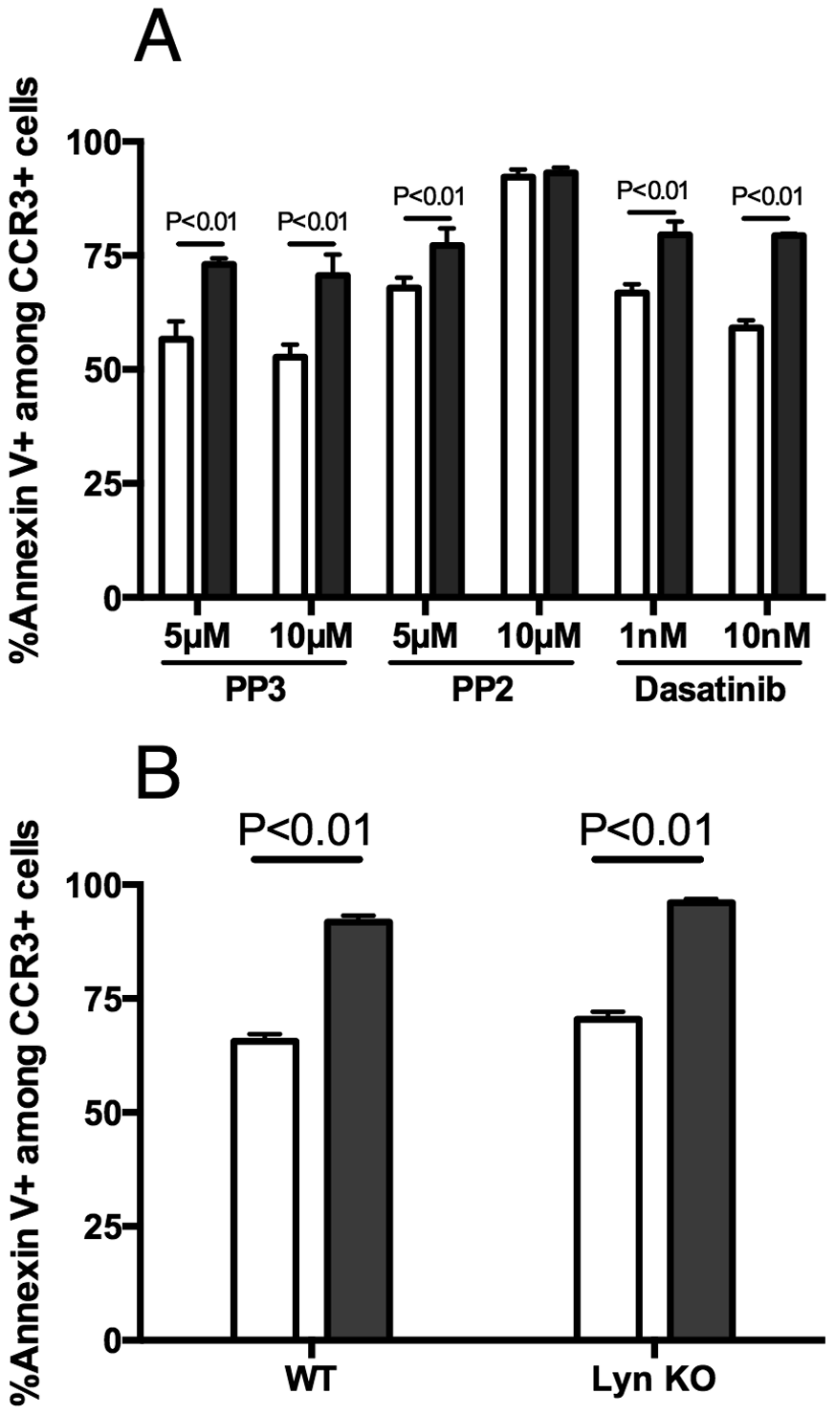
Src family kinases are not required for Siglec-F-induced eosinophil cell death. Eosinophils from wild type (panel A) and Lyn-deficient (Lyn KO, panel B) mice were treated with anti-Siglec-F (filled bars) or isotype-matched control antibody (open bars). In panel A, eosinophils from peritoneal lavage were treated in the presence of PP2 (5 and 10 µM) and dasatinib (1 and 10 nM). Shown are data from a single experiment representative of n = 3 experiments with similar results. In panel B, eosinophils were obtained from a Lyn deficient mouse by peritoneal lavage (n = 1, shown as the representative experiment, to match the cell source of panel A) or from mouse bone marrow-derived eosinophils (n = 3, data not shown but with similar results). Error bars in these representative experiments represent the standard deviation of triplicates.

Because the ITIM and ITIM-like domains may have functions independent of SHP-1 activation, we tested the role of Src family kinases that have been shown to phosphorylate ITIM/ITIM-like domains of inhibitory receptors, including siglecs. As seen in Figure 4A, inhibition of Src family kinases with the compound PP2 in the absence of anti-Siglec-F (open bars) led to increased eosinophil cell death compared to eosinophils incubated with inactive control compound PP3, suggesting that Src family kinases are required for eosinophil viability in general. However, Siglec-F-induced eosinophil cell death (filled bars) was comparable in cells incubated with and without Src-family inhibitor PP2 as well as a broader kinase inhibitor dasatinib, suggesting that Src family kinases are not required for Siglec-F-induced cell death. Furthermore, we used an independent genetic approach to assess specifically the role for Lyn kinase, and found that Lyn-deficient eosinophils underwent eosinophil cell death at levels comparable to that of wild type cells (Figure 4B). Together, these data demonstrate that Src kinases are not required for Siglec-F-induced eosinophil cell death.

**Figure 5:**
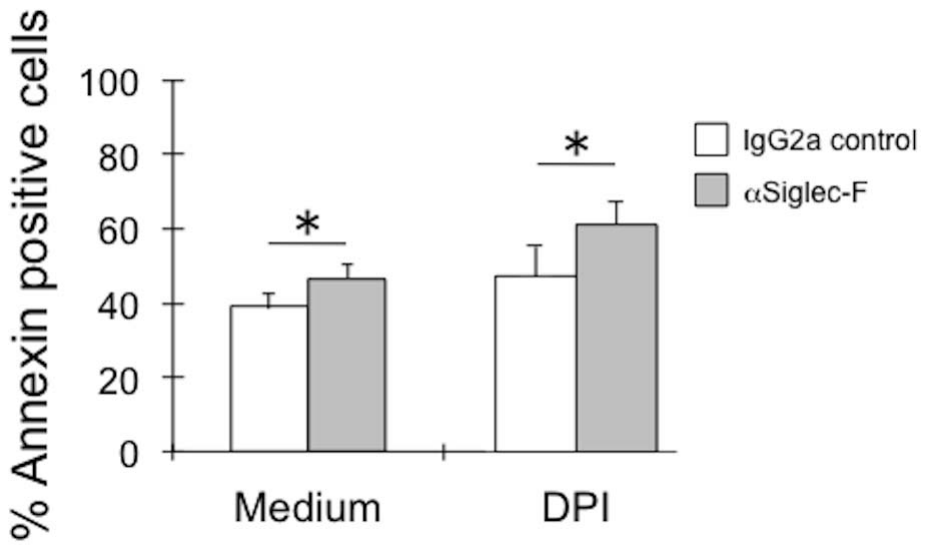
Effects of DPI on Siglec-F-induced apoptosis of eosinophils. Wild-type mouse bone marrow-derived eosinophils (n = 4) were incubated with or without DPI (20 µM) for 5 min before adding either a control mAb or Siglec-F mAb and then cells were cultured in IL-5 for an additional 18 hr before determining Annexin positivity. * p<0.05.

Lastly, mouse eosinophils were tested to determine whether Siglec-F engagement might trigger generation of ROS within mitochondria (perhaps at low-levels), and whether mitochondria-derived ROS might be necessary or sufficient for Siglec-F-induced apoptosis. For these experiments, a pharmacologic inhibitor of ROS production was employed. As shown in Figure 5, the mitochondrial electron transport inhibitor, DPI, did not prevent Siglec-F-induced apoptosis. This was in contrast to prior studies in human eosinophils, where DPI completely blocked Siglec-8-induced apoptosis of cytokine-primed human eosinophils [26].

## Discussion

In this paper, we have found that there is no detectable role for ROS in Siglec-F-mediated apoptosis. This conclusion was based on an inability to detect any ROS production after Siglec-F mAb exposure in normal or IL-5 primed mouse eosinophils derived from blood, spleen or culture, and similar rates of apoptosis using eosinophils derived from Ncf^−/−^ mice deficient in NADPH oxidase activity. Instead, Siglec-F-mediated apoptosis was completely blocked by the addition of a broad-spectrum caspase inhibitor. While the latter has also been reported to play a role in Siglec-8 function in non-cytokine primed human eosinophils [22], [25], the lack of a ROS-dependent pathway for Siglec-F-induced eosinophil death, even after cytokine activation, represents a difference between Siglec-F signaling in mouse eosinophils and Siglec-8 signaling in human eosinophils. Despite these differences, it remains to be determined how Siglec-F, which lacks any classical motifs for caspase activation, initiates this death pathway.

In additional experiments to explore the potential involvement of the Siglec-F cytoplasmic ITIM domain using eosinophils derived from me^v^ mice deficient in SHP-1 function, it was observed that SHP-1 was not required for Siglec-F-induced eosinophil apoptosis. While one cannot rule out the possibility that other tyrosine phosphatases may be involved in Siglec-F or Siglec-8-mediated signaling, most prior reports of signaling via CD33-family siglecs have implicated SHP-1 [8], [29], [33], [34]. Since the ITIM motif is often phosphorylated by Src family kinases (which leads to binding of phosphatases such as SHP-1, or other signal transduction molecules), we tested the role of Src family kinases and specifically Lyn kinase. However, our data suggest Src family kinases are not involved in the apoptosis induced by Siglec-F. Our recent studies have implicated MAP kinases ERK-1/2 in Siglec-8-mediated human eosinophil cell death (Kano et al. Mechanism of Siglec-8-mediated cell death in IL-5-activated eosinophils: Role for reactive oxygen species-enhanced MEK/ERK activation. J. Allergy Clin. Immunol. in press), and thus future studies will assess the role of this pathway in Siglec-F-mediated cell death of mouse eosinophils. Given the presence of the membrane distal ITSM motif conserved in both Siglec-8 and Siglec-F, it is also possible that there is a differential role of this cytoplasmic region in different cell types. Indeed, in human mast cells the membrane proximal ITIM motif completely controls the inhibitory effect of Siglec-8 engagement on FcεRI-mediated degranulation, yet these cells fail to undergo Siglec-8-dependent apoptosis [46]. More work is needed to explore the exact role of the membrane distal ITSM motif in signaling via Siglec-F and Siglec-8.

In conclusion, the work reported here has uncovered potential differences between Siglec-F and Siglec-8 signaling and its consequences. Despite the remarkably consistent benefits of targeting Siglec-F in mouse models of hypereosinophilia, asthma and gastrointestinal eosinophilia and the exaggerated eosinophilic responses seen in mice deficient in Siglec-F when put through various models of allergic inflammation [12], based on the data presented herein, Siglec-F appears to have a different intracellular mechanism of activity than Siglec-8 in that even under cytokine priming conditions, cell death is always caspase dependent. Given the fact that Siglec-F is a functional paralog rather than a true ortholog of Siglec-8 [17] and that below chimpanzees there is no Siglec-8 ortholog [47], [48] efforts to advance the targeting of Siglec-8 as a potential therapeutic for human diseases will need to consider other strategies besides simply relying on data from studies of Siglec-F.
